# Prognostic value of *maspin* mRNA expression in ER*α*-positive postmenopausal breast carcinomas

**DOI:** 10.1038/sj.bjc.6600812

**Published:** 2003-03-18

**Authors:** I Bièche, I Girault, J-C Sabourin, S Tozlu, K Driouch, M Vidaud, R Lidereau

**Affiliations:** 1 Laboratoire d'Oncogénétique, INSERM E0017, 35 rue Dailly, Centre René Huguenin, F-92211 St Cloud, France; 2 Laboratoire de Génétique Moléculaire, UPRES EA 3618, Faculté des Sciences Pharmaceutiques et Biologiques, Université René Descartes, Paris V, 4 Avenue de l'Observatoire, F-75006 Paris, France; 3 Laboratoire d'Anatomo-Cytopathologie, 35 rue Dailly, Centre René Huguenin, F-92211 St Cloud, France

**Keywords:** breast cancer, *maspin* gene expression, real-time RT–PCR quantification, prognostic value

## Abstract

Maspin, a member of the serpin family, has a role in cell migration, angiogenesis and apoptosis. Little is known of the clinical significance of *maspin* gene expression in human cancers. We developed a real-time quantitative RT–PCR assay to quantify the full range of *maspin* mRNA copy numbers in a series of 10 ER*α*-positive and 10 ER*α*-negative breast tumours. We observed a statistical link between low *maspin* mRNA levels and positive oestrogen status (*P*=0.0012). In consequence, to better assess the prognostic value of *maspin* gene expression in breast cancer, we then quantified *maspin* mRNA content in an additional independent well-defined cohort of 105 ER*α*-positive postmenopausal breast cancer patients treated with primary surgery followed by adjuvant tamoxifen alone. *Maspin* expression varied widely in tumour tissues (by nearly four orders of magnitude), being underexpressed in 33 out of 105 tumours (31.4%) and overexpressed in 24 out of 105 tumours (22.9%) relative to normal breast tissues. Immunohistochemical studies demonstrated that maspin protein was strictly expressed in myoepithelial cells of normal breast tissue and in tumour epithelial cells, exclusively in maspin-overexpressing tumours. Patients with tumours overexpressing the *maspin* gene had significantly shorter relapse-free survival after surgery than patients whose tumours normally expressed or underexpressed *maspin* (*P*=0.0011). The prognostic significance of *maspin* overexpression persisted in Cox multivariate regression analysis (*P*=0.0024). These findings show that the *maspin* mRNA level can have important prognostic significance in human breast cancer, and point to the *maspin* gene as a putative molecular predictor of hormone responsiveness in breast cancer.

Maspin (mammary serpin) is a 42-kDa cytoplasmic protein belonging to the serpin family of serine protease inhibitors (Zou *et al*, 1994). The *maspin* gene was originally identified in normal mammary epithelium by subtractive hybridisation on the basis of its expression at the mRNA level (Zou *et al*, 1994). Maspin has subsequently been localised to epithelial and myoepithelial cells in a variety of tissues (Pemberton *et al*, 1997).

Biological studies suggest a tumour-suppressive role of maspin, mediated by effects on cell migration (Zou *et al*, 1994; Sheng *et al*, 1996), angiogenesis (Zhang *et al*, 2000a) and apoptosis (Zhang *et al*, 2000b; Jiang *et al*, 2002). Maspin expression in breast tumour cells reduces tumour induction and metastasis in nude mice (Zou *et al*, 1994; Shi *et al*, 2001; Streuli, 2002), and also invasion of the basement membrane *in vitro* (Zou *et al*, 1994). Moreover, treatment of human breast cancer cells with recombinant maspin (rMaspin) inhibits cell motility (Sheng *et al*, 1996). Possible molecular targets for maspin are tissue-type plasminogen activator (tPA) (Sheng *et al*, 1998) and various integrins (Seftor *et al*, 1998).

The role of maspin in human breast cancer is poorly documented, and even less is known about the clinical significance of maspin expression in this setting. The few available data on maspin expression in breast cancer have been obtained using breast cancer cell lines and breast tumour series (Zou *et al*, 1994; Jiang *et al*, 1997; Domann *et al*, 2000; Maass *et al*, 2001a), and the results are confusing. Indeed, maspin expression is generally downregulated in primary breast tumours and lost in mammary carcinoma lines (Zou *et al*, 1994; Hojo *et al*, 2001; Maass *et al*, 2001a,2001b), but some infiltrating breast tumours have been found to show strong upregulation (Hojo *et al*, 2001; Maass *et al*, 2001b). Maspin expression has been found to decrease with increasing malignancy in primary breast tumours, and to be absent from distant metastases (Zou *et al*, 1994). Surprisingly, Martin *et al* (2000), using cDNA array technology, identified the *maspin* gene as the leader of a cluster of genes that are strongly upregulated in ER*α*-negative breast tumours (ER*α* negativity is associated with aggressive tumours). Finally, four recent clinical studies of heterogeneous series of women with breast tumours examined the possible association between maspin expression (mRNA or protein level) and classical clinical and pathological parameters, including patient outcome (Hojo *et al*, 2001; Maass *et al*, 2001a,2001b). The results of these studies are conflicting, three studies suggested that *maspin* underexpression could be a potential poor prognostic marker in breast cancer, while the fourth identified *maspin* overexpression rather as an independent poor prognostic indicator in breast cancer patients (Umerika *et al*, 2002).

Here, we developed a real-time quantitative RT–PCR assay to quantify *maspin* mRNA in homogeneous total RNA solutions obtained from human tissue samples. As we found that *maspin* gene expression was strongly linked to ER*α* expression status, we quantified maspin mRNA expression in a well-defined cohort of 105 ER*α*-positive postmenopausal breast cancer patients who were treated with primary surgery, followed by adjuvant tamoxifen alone and whose long-term outcome was known. The significance of *maspin* as a prognostic factor is discussed.

## MATERIALS AND METHODS

### Patients and samples

We analysed tissue samples from primary breast tumours excised from 105 women at Centre René Huguenin from 1980 to 1994. Tumour tissue samples of the 105 patients were collected in accordance with French regulations.

Immediately following surgery, the tumour samples were stored in liquid nitrogen until RNA extraction. The patients (mean age 70.8 years, range 54–86) met the following criteria: primary unilateral nonmetastatic postmenopausal breast carcinoma; oestrogen receptor alpha positivity (as determined at the protein level by biochemical methods (dextran-coated charcoal method until 1988 and thereafter EIA) and confirmed by *ERα* real-time quantitative RT–PCR assay); complete clinical, histological and biological information available; no radiotherapy or chemotherapy before surgery; and full follow-up at Centre René Huguenin. Among the 105 breast tumours, 97 were invasive ductal carcinomas and eight were invasive lobular carcinomas. The standard prognostic factors are presented in Table 1

**Table 1:** Characteristics of the 105 ER*α*-positive postmenopausal patients and their relation to RFS

		**RFS**	
	**Number of patients**	**Number of events (%)^a^**	***P*-value^b^**
*Age (years)*			NS
⩽70	52	17 (32.7)	
>70	53	14 (26.4)	
			
*Histological grade*^c^			0.00067
I+II	80	17 (21.2)	
III	24	13 (54.2)	
			
*Lymph node status*			0.003
0	17	2 (11.8)	
1–3	59	14 (23.7)	
>3	29	15 (51.7)	
			
*Macroscopic tumour size (mm)*			0.021
⩽30	73	17 (23.3)	
>30	32	14 (43.7)	
			
*Maspin RNA status*			0.0011
Underexpressed	33	7 (21.2)	
Normal	48	11 (22.9)	
Overexpressed	24	13 (54.2)	

a First relapses (local and/or regional recurrences, and/or distant metastases).

b Log-rank test. NS=not significant.

c Scarff–Bloom–Richardson classification. Information available for 104 patients.

. A total of 34 patients (32.4%) had modified radical mastectomy and 71 (67.6%) had breast-conserving surgery plus locoregional radiotherapy. Patients underwent physical examinations and routine chest radiography every 3 months for 2 years, then annually. Mammograms were performed annually. The median follow-up was 6.0 years (range 1.5–10.0 years). All the patients received postoperative adjuvant endocrine therapy (tamoxifen, 20 mg daily for 3–5 years), and no other treatment. In all, 31 patients relapsed (the distribution of first relapse events was as follows: 27 metastases, and four both local and/or regional recurrences and metastases).

To investigate the relation between *maspin* mRNA levels and ER*α* expression status, we also analysed 20 additional primary breast tumours: 10 ER*α*-negative and 10 ER*α*-positive tumours.

We analysed six breast tumuor cell lines obtained from the American Tissue Type Culture Collection (SK-BR-3, T-47D, BT-20, HBL-100, ZR-75-1 and MCF7).

Specimens of adjacent normal breast tissue from five of the breast cancer patients, and normal breast tissue from three women undergoing cosmetic breast surgery, were used as sources of normal RNA.

### Real-time RT–PCR

#### Theoretical basis

Reactions are characterised by the point during cycling when amplification of the PCR product is first detected, rather than the amount of PCR product accumulated after a fixed number of cycles. The higher the starting copy number of the target transcript, the earlier a significant increase in fluorescence is observed. The parameter *C*_t_ (threshold cycle) is defined as the fractional cycle number at which the fluorescence generated by the incorporation of a fluorogenic molecule in double-stranded DNA passes a fixed threshold above baseline.

The precise amount of total RNA added to each reaction mix (based on optical density) and its quality (i.e. lack of extensive degradation) are both difficult to assess. We therefore also quantified transcripts of the gene coding for the TATA box-binding protein (*TBP*) (a component of the DNA-binding protein complex TFIID) as the endogenous RNA control, and each sample was normalised on the basis of its *TBP* content.

The relative *maspin* gene expression level was also normalised to a calibrator, or 1 × sample, consisting of a pool of normal breast tissue specimens.

Final results, expressed as *N*-fold differences in *maspin* gene expression relative to the *TBP* gene and normal breast tissues (the calibrator), termed ‘*N*_*maspin*_’, were determined as follows:

*N*_*maspin*_ = 2^(Δ*C*_*t sample*_ − Δ*C*_*t calibrator*_)^ where Δ*C*_t_ values of the sample and calibrator are determined by subtracting the average *C*_t_ value of the *maspin* gene from the average *C*_t_ value of the *maspin* gene.

#### Primers and PCR consumables

Primers for the *TBP* and *maspin* genes were chosen with the assistance of the computer programs Oligo 4.0 (National Biosciences, Plymouth, MN, USA). We conducted BLASTN searches against dbEST, htgs and nr (the nonredundant set of the GenBank, EMBL and DDBJ database sequences) to confirm the total gene specificity of the nucleotide sequences chosen for the primers, and the absence of DNA polymorphisms. The nucleotide sequences of the primers used were as follows: Maspin-U (5′-CTA CTT TGT TGG CAA GTG GAT GAA-3′) and Maspin-L (5′-ACT GGT TTG GTG TCT GTC TTG TTG-3′) for *maspin* gene (PCR product of 90 bp), and TBP-U (5′-TGC ACA GGA GCC AAG AGT GAA-3′) and TBP-L (5′-CAC ATC ACA GCT CCC CAC CA-3′) for *TBP* gene (PCR product of 132 bp).

To avoid amplification of contaminating genomic DNA, one of the two primers was placed in a different exon. For example, the upper primer of *TBP* was placed at the junction between exons 5 and 6, whereas the lower primer was placed in exon 6. Agarose gel electrophoresis allowed us to verify the specificity of PCR amplicons.

#### RNA extraction

Total RNA was extracted from breast specimens by using the acid-phenol guanidium method. The quality of the RNA samples was determined by electrophoresis through agarose gels and staining with ethidium bromide, and the 18S and 28S RNA bands were visualised under ultraviolet light.

#### cDNA synthesis

RNA was reverse transcribed in a final volume of 20 *μ*l containing 1 × RT buffer (500 mM each dNTP, 3 mM MgCl_2_, 75 mM KCl, 50 mM Tris-HCl pH 8.3), 10 units of RNasinTM Ribonuclease inhibitor (Promega, Madison, WI, USA), 10 mM dithiothreitol, 50 U of Superscript II RNase H-reverse transcriptase (Gibco BRL, Gaithersburg, MD, USA), 1.5 mM random hexamers (Pharmacia, Uppsala, Sweden) and 1 *μ*g of total RNA. The samples were incubated at 20°C for 10 min and 42°C for 30 min, and reverse transcriptase was inactivated by heating at 99°C for 5 min and cooling at 5°C for 5 min.

#### PCR amplification

All PCR reactions were performed using an ABI Prism 7700 Sequence Detection System (Perkin-Elmer Applied Biosystems, Foster City, CA). Polymerase chain reaction was performed using the SYBR® Green PCR Core Reagents kit (Perkin-Elmer Applied Biosystems, Foster City, CA). The thermal cycling conditions comprised an initial denaturation step at 95°C for 10 min and 50 cycles at 95°C for 15 s and 65°C for 1 min. Experiments were performed with duplicates for each data point.

### Immunohistochemical studies

Indirect immunoperoxidase staining of fixed tissues was performed using mouse monoclonal antibody G167-70 directed against the human maspin protein (PharMingen, San Diego, CA) and monoclonal antibody SMMS-1 raised against human myosin heavy chain (Dakocytomation Denmark A/S, Glostrup, DK).

The immunohistochemical procedure was applied to paraffin-embedded tissue sections. A water bath antigen-retrieval technique was used in all cases. Sections were mounted on precoated slides (Dako) and allowed to dry at 50°C overnight. The sections were then dewaxed in xylene and hydrated through graded dilutions of ethanol. Endogenous activity was blocked with 1% hydrogen peroxide for 15 min. Sections were then immersed in a heat-resistant plastic box containing 10 ml of pH 6.0 citrate buffer and processed in the water bath for 40 min. Sections were then allowed to cool to room temperature for 20 min before rinsing in H_2_O. The blocking reagent was poured off and the primary antibodies were left for 25 min. A standard avidin–biotin–peroxidase complex (LSAB) method was used to reveal the antibody–antigen reaction (Dako).

The localisation and intensity of staining were assessed by two independent pathologists who were blinded to the real-time RT–PCR results.

### Statistical analysis

Relapse-free survival (RFS) was determined as the interval between diagnosis and detection of the first relapses (local and/or regional recurrences, and/or metastases).

Clinical, histological and biological parameters were compared using the *χ*^2^ test. Differences between the two populations were judged significant at confidence levels greater than 95% (*P*<0.05). The relation between *maspin* mRNA levels and ER*α* status was assessed using the Kruskal–Wallis test. Survival distributions were estimated by the Kaplan-Meier method (Kaplan and Meier, 1958), and the significance of differences between survival rates was ascertained using the log-rank test (Peto *et al*, 1977). Cox's proportional hazards regression model (Cox, 1972) was used to assess prognostic significance.

## RESULTS

### *Maspin* mRNA expression in normal breast tissues

To determine the cutoff point for altered *maspin* expression in breast cancer tissue, the *N*_*maspin*_ value, calculated as described in Patients and Samples, was determined for eight normal breast RNA samples (Table 2

**Table 2:** *Maspin* gene expression in human breast tissues

**Sample no.**	***N*_*maspin*_ value**
*Normal breast tissues*	
P1	0.93
P2	1.06
P3	1.17
P4	1.35
P5	0.73
P6	0.59
P7	0.84
P8	1.67
	
Mean (s.d.)	1.04±0.35
	
*ERα-negative breast tumours*	
T1	1.62
T2	5.22
T3	3.92
T4	40.3
T5	1.51
T6	3.33
T7	96.8
T8	2.69
T9	1.33
T10	0.89
	
Mean (s.d.)	15.76±30.87
	
*ERα-positive breast tumours*	
T1	0.78
T2	0.02
T3	0.72
T4	3.89
T5	0.33
T6	0.24
T7	0.47
T8	0.17
T9	0.84
T10	1.26
Mean (s.d.)	0.87±1.12

). As this value consistently fell between 0.59 and 1.67 (mean 1.04±s.d. 0.35), values of 3 (mean±5 s.d.) or more were considered to represent marked overexpression, and values of 0.30 (mean−2 s.d.) or less were considered to represent underexpression of *maspin* mRNA. We have previously used the same approach to determine the cutoff points for altered expression of other tumour genes (Bièche *et al*, 1999a).

### Relation between *maspin* mRNA levels and *ERα* expression status

Maspin mRNA levels were determined in 10 ER*α*-negative and 10 selected ER*α*-positive breast tumours with very high ER*α* mRNA values (Table 2). These 10 ER*α*-negative and 10 ER*α*-positive breast tumours have been selected from a previously tested breast tumours series where the ER*α* status was determined at the protein level by biochemical methods and quantified by *ERα* real-time quantitative RT–PCR assay (Bièche *et al*, 2001).

We found a negative correlation between *maspin* and ER*α* mRNA expression (*P*=0.0012): the 10 ER*α*-negative breast tumours had higher levels of *maspin* mRNA (0.89- to 96.8-fold normal; mean 15.76±s.d. 30.87) than the 10 ER*α*-positive breast tumours (0.02- to 3.89-fold; mean 0.87±s.d. 1.12) (Table 2).

### *Maspin* mRNA expression in a well-defined set of 105 ER*α*-positive postmenopausal breast tumour RNA samples

To better assess the prognostic value of *maspin* gene expression in ER*α*-positive breast cancer, we quantified *maspin* mRNA content in an additional independent well-defined cohort of 105 ER*α*-positive postmenopausal breast cancer patients treated with primary surgery followed by adjuvant tamoxifen alone. These breast tumour RNA samples had a wide range of *N*_*maspin*_ values (0.01–46.7, i.e. nearly four orders of magnitude).

Compared to normal breast tissues, 57 (54.3%) tumours showed altered *maspin* mRNA expression. A total of 33 tumours (31.4%) showed *maspin* mRNA underexpression (*N*_*maspin*_ from 0.01 to 0.28) and 24 (22.9%) showed *maspin* mRNA overexpression (*N*_*maspin*_ from 3.11 to 46.7).

*maspin* expression was also investigated in six breast tumour cell lines. All showed *maspin* underexpression, with *N*_*maspin*_ values of 0.004 (SK-BR-3), 0.029 (T-47D), 0.045 (BT-20), 0.001 (HBL-100), 0.042 (ZR-75-1) and 0.017 (MCF7).

### Localisation of *maspin* protein in epithelial tumour cell cytoplasm

**Figure 1:**
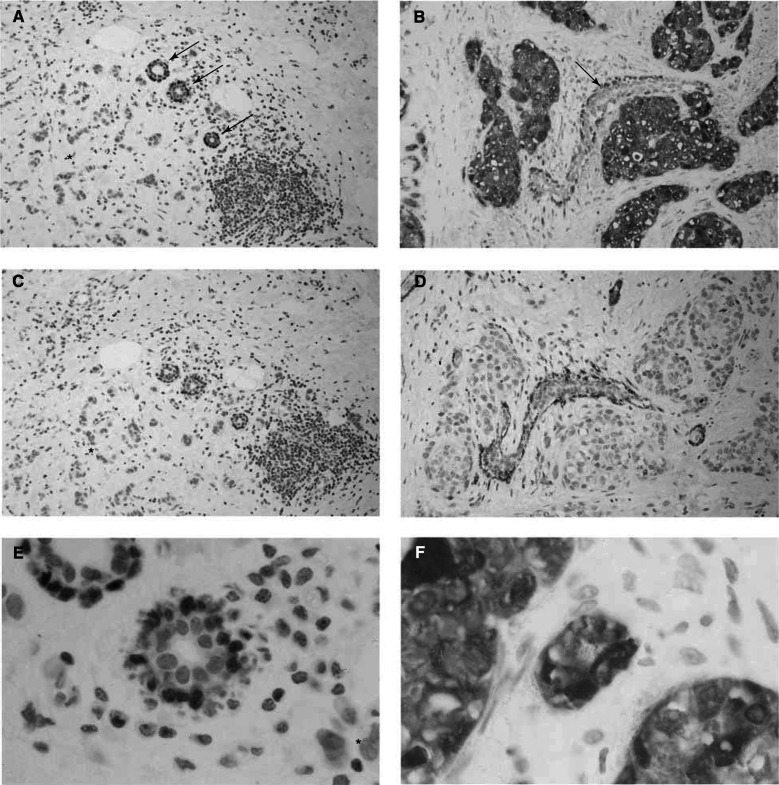
Immunohistochemical staining for maspin (**A**, **B**, **E**, **F**) and for myosin heavy chain (**C**, **D**) in two breast tumours, one with normal *maspin* expression (**A**, **C**, **E**) and one with *maspin* overexpression (**B**, **D**, **F**). Intense *maspin* immunoreactivity was found in tumour epithelial cells from the *maspin* mRNA-overexpressing tumour (**B**) but not in cells (star) from the tumour without *maspin* overexpression (**A**) (original magnification × 20). Note the negative myosin heavy-chain staining of tumour epithelial cells from the *maspin* mRNA-overexpressing tumour (**D**), confirming that the latter is not of myoepithelial origin (original magnification × 20). In both cases, normal breast tissue showed *maspin* immunoreactivity in normal myoepithelial cells (arrows) (**A**, **B**), which also showed strong immunoreactivity for myosin heavy chain (**C**, **D**) (original magnification × 20). Maspin immunoreactivity was localised in the cytoplasmic compartment of both epithelial tumour cells (**F**) and normal myoepithelial cells (**E**) (original magnification × 100).

We detected specific maspin immunoreactivity in normal myoepithelial cells of all eight tumour samples studied by IHC. We also detected strong specific immunoreactivity in epithelial cells of the four tumours which overexpressed *maspin* mRNA and in none of the four tumours which did not overexpress *maspin*. We thus obtained a perfect match between *maspin* mRNA overexpression and IHC positivity (Figure 1

). Maspin immunoreactivity was exclusively found in normal myoepithelial cells and tumour epithelial cells; infiltrating lymphocytes and normal glandular cells in the tumour were consistently negative. Staining was found exclusively in the cytoplasm of both normal myoepithelial and tumour epithelial cells (Figure 1). The positive tumour epithelial cells were myosin heavy-chain-negative (Figure 1), and were therefore not of myoepithelial origin.

### Correlation between *maspin* mRNA levels and clinical, pathological and biological parameters

We sought links between *maspin* mRNA expression status and standard clinical and pathological factors in breast cancer (Table 3

**Table 3:** Relation between *maspin* mRNA level and the standard clinical, pathological and biological factors

		***maspin* mRNA level**			
		**Number of patients (%)**			
	**Total population (%)**	**Underexpression**	**Normal**	**Overexpression**	***P*-value^a^**
Total	105 (100.0)	33 (31.4)	48 (45.7)	24 (22.9)	
					
*Age (years)*					NS
⩽70	52 (49.5)	15 (45.5)	27 (56.2)	10 (41.7)	
70	53 (50.5)	18 (54.5)	21 (43.8)	14 (58.3)	
					
*Histological grade*^b^,^c^					0.03
I+II	80 (76.9)	27 (81.8)	40 (83.3)	13 (56.5)	
III	24 (23.1)	6 (18.2)	8 (16.7)	10 (43.5)	
					
*Lymph node status*					NS
0	17 (16.2)	5 (15.2)	7 (14.6)	5 (20.8)	
1–3	59 (56.2)	15 (45.4)	32 (66.7)	12 (50.0)	
>3	29 (27.6)	13 (39.4)	9 (18.7)	7 (29.2)	
					
*Macroscopic tumour size (mm)*					NS
⩽30	73 (69.5)	22 (66.7)	34 (70.8)	17 (70.8)	
>30	32 (30.5)	11 (33.3)	14 (29.2)	7 (29.2)	
					
*Histological type*					NS
Ductal	97 (92.4)	32 (97.0)	42 (87.5)	23 (95.8)	
Lobular	8 (7.6)	1 (3.0)	6 (12.5)	1 (4.2)	
					
ERBB2 *RNA status*					NS
Overexpressed	18 (17.1)	3 (9.1)	8 (16.7)	7 (29.2)	
Normal	87 (82.9)	30 (90.9)	40 (83.3)	17 (70.8)	
					
ERβ *RNA status*^c^					NS
Low	36 (34.6)	14 (42.4)	15 (31.9)	7 (29.2)	
Intermediate	34 (32.7)	7 (21.2)	17 (36.2)	10 (41.6)	
High	34 (32.7)	12 (36.4)	15 (31.9)	7 (29.2)	

a *χ*^2^-test.

b Scarff–Bloom–Richardson classification.

c Information available for 104 patients.

). The only statistically significant association (*χ*^2^ test) was between *maspin* mRNA overexpression and Scarff–Bloom–Richardson (SBR) histopathological grade III (*P*=0.03). *maspin* mRNA status was not significantly associated with age, lymph node status, macroscopic tumour size or histological type.

We also found no link between *maspin* mRNA status and the mRNA status of two candidate genes predicting the response to endocrine therapy, namely *ERBB2* and *ERβ* (estrogen receptor*β*) (Table 3).

### Prognosis analysis of *maspin* mRNA expression

**Figure 2:**
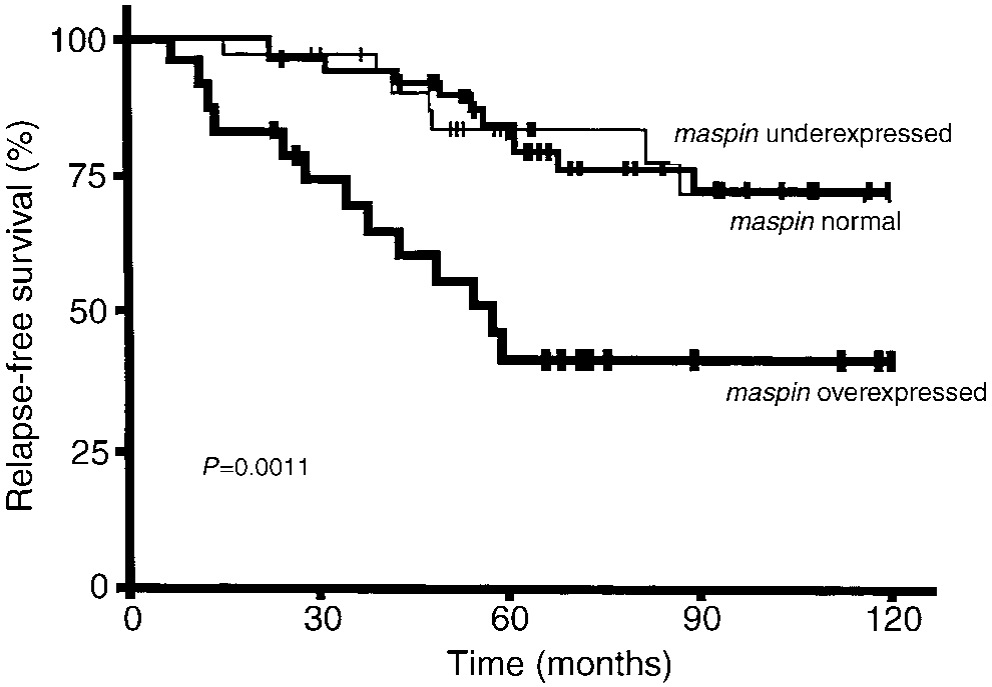
Relapse-free survival curves for patients with *maspin*-overexpressing, normally expressing and underexpressing tumours.

Univariate analysis (log-rank test) showed that RFS was linked to *maspin* mRNA status (*P*=0.0011; Figure 2

). The RFS of the 24 patients with *maspin*-overexpressing tumours (5-year RFS 41.8±10.5%) was shorter than that of the 48 patients whose tumours normally expressed *maspin* (5-year RFS 84.4±5.4%) and also shorter than that of the 33 patients with *maspin*-underexpressing tumours (5-year RFS 83.6±6.7%).

**Figure 3:**
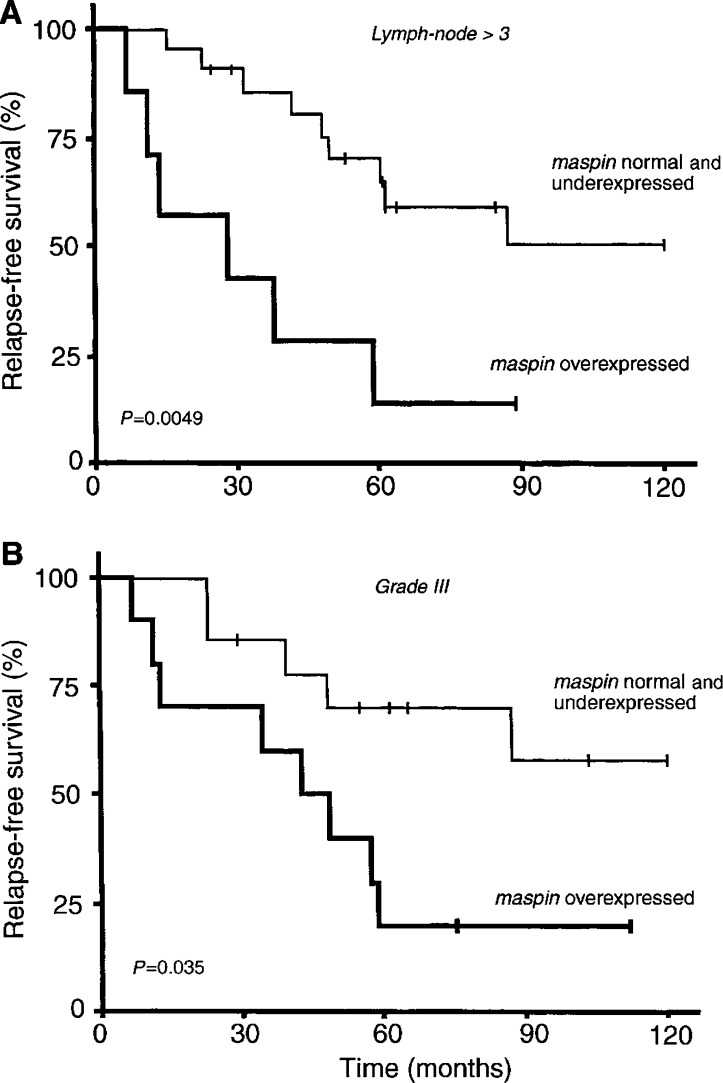
Relapse-free survival curves in poor-prognosis patient subpopulations (involved lymph nodes >3 (**A**) and histopathological grade III (**B**)) according to whether their tumours overexpressed *maspin* or showed normal/low *maspin* expression.

Interestingly, the outcome of the patients with *maspin*-overexpressing tumours was also significantly worse when the analysis focused on two well-known specific subsets of poor-prognosis breast cancer patients, that is, those with more than three involved lymph nodes (*P*=0.0049, Figure 3A

) and those with histopathological grade III (*P*=0.035, Figure 3B).

Finally, the prognostic significance of the four parameters identified in univariate analysis (histopathological grade III, lymph-node status, macroscopic tumour size (Table 1) and *maspin* overexpression status (Table 1, Figure 2)) persisted (except for macroscopic tumour size) in Cox multivariate regression analysis of RFS (Table 4

**Table 4:** Multivariate analysis of RFS

	**RFS**		
**Variables**	**Regression coefficient**	**Relative risk (95% CI)^a^**	***P*-value**
*Lymph node status*			0.0014
0	1	1	
1–3	0.99	2.69 (1.46–4.94)	
>3	1.98	7.23 (2.14–24.40)	
			
maspin *mRNA status*			0.0024
Underexpression/normal	1	1	
Overexpression	1.23	3.43 (1.55–7.62)	
			
*Histological grade*			0.011
I+II	1	1	
III	0.96	2.61 (1.25–5.46)	

a 95% confidence interval.

).

## DISCUSSION

Since 1994, when *maspin* was first described as a tumour-suppressor gene in breast cancer, little has been learnt about its expression. Maspin protein is present in the breast epithelium, and particularly in the myoepithelium, and *maspin* expression has been reported to be both decreased and increased in subsets of breast tumours, and lost in some metastases (Zou *et al*, 1994; Jiang *et al*, 1997; Domann *et al*, 2000). Four clinical studies of *maspin* expression have recently been reported, and these mainly focused on underexpression status, one at the mRNA level (Maass *et al*, 2001a) and three at the protein level (Hojo *et al*, 2001; Maass *et al*, 2001b; Umerika *et al*, 2002). Maass *et al* (2001a), using a semiquantitative nested RT–PCR assay in a small series of primary breast carcinoma tissues (*n*=45) with short follow-up (3 years), showed no maspin transcripts in 16 (36%) breast tumours. Maspin mRNA expression was unrelated to established prognostic factors, including ER*α* status, but six of the eight patients who developed distant metastasis within 3 years of the initial diagnosis showed no *maspin* mRNA expression in their primary breast tumours. Two immunochemical studies, although involving heterogeneous breast tumours series with no follow-up, also suggest that *maspin* underexpression could be a marker of breast cancer aggressiveness (Hojo *et al*, 2001; Maass *et al*, 2001b). In contrast, the third study identified *maspin* overexpression as an independent poor prognostic indicator in breast cancer patients (Umerika *et al*, 2002).

We used real-time quantitative RT–PCR to assess *maspin* gene expression in human breast tumours. This recent approach to nucleic acid quantification is suited to the development of target gene assays, having a high degree of inter-laboratory standardisation and yielding statistical confidence values (Bièche *et al*, 1999b). The main advantage of real-time RT–PCR is its large linear dynamic range. This made it particularly appropriate for analysing *maspin* gene expression, which ranged from 0.01 to 46.7 times normal in this series. Contrary to Maass *et al* (2001a), who failed to detect *maspin* mRNA in 36% of their breast cancer specimens with a semiquantitative nested RT–PCR assay, we detected *maspin* mRNA in all the breast tumour samples tested, probably reflecting the higher sensitivity of our real-time RT–PCR method.

It has been suggested that *maspin* dysregulation in tumours may be because of altered expressions of various transregulators acting on the *maspin* promoter, including the proteins Ets and Ap1 (Zhang *et al*, 1997) and p53 (Zou *et al*, 2000). A recent publication also discussed the involvement of aberrant cytosine methylation and chromatin condensation of the *maspin* promoter in the silencing of *maspin* expression during neoplastic progression (Domann *et al*, 2000).

We found a strong negative correlation between *maspin* gene expression and ER*α* expression status (Table 2), in agreement with Umerika *et al* (2002) and Martin *et al* (2000). These latter authors, using cDNA array technology, identified *maspin* gene as the leader of a cluster of genes that are strongly upregulated in ER*α*-negative breast tumours. Thus, to test the prognostic value of *maspin* mRNA expression in breast cancer, we chose to analyse a well-defined cohort of 105 postmenopausal patients with ER*α*-positive breast cancer treated with primary surgery followed by adjuvant tamoxifen alone.

We observed both underexpression (31.4% of samples) and overexpression (22.9%) of *maspin* mRNA.

In all, 33 tumours, and all six breast tumour cell lines tested (SK-BR-3, 47D, BT-20, HBL-100, ZR-75-1 and MCF7) underexpressed *maspin* mRNA. These results are in agreement with reports from several authors (Zou *et al*, 1994; Domann *et al*, 2000; Maass *et al*, 2001a) showing *maspin* underexpression at the RNA and/or protein level in breast cancer, and corroborating biological evidence that *maspin* is a tumour-suppressor gene. The very low maspin mRNA values in the six breast tumour cell lines tested by real-time RT–PCR were in total agreement with previous data (Zou *et al*, 1994; Zhang *et al*, 1997; Domann *et al*, 2000; Maass *et al*, 2001a). These very low values observed in the tumour cell lines could be because of the depletion in the *in vitro* epithelial breast tumour cell cultures of the myoepithelial normal cells, which secrete high levels of maspin (Sternlicht and Barsky, 1997). Contrary to Maass *et al* (2001a), but in agreement with Martin *et al* (2000) and Umerika *et al* (2002), we found an association between *maspin* mRNA underexpression and oestrogen receptor alpha positivity, suggesting that *maspin* mRNA underexpression is associated with low tumour aggressiveness (Table 2).

Finally, RFS after surgery was not shorter in patients whose tumours underexpressed *maspin* than in patients whose tumours normally expressed *maspin* (Figure 2). Zou *et al* (1994) and Sheng *et al* (1996) reported that maspin inhibited the invasive and metastatic potential of breast cancer cells, while Maass *et al* (2001a) found that *maspin* downregulation was associated with a higher risk of early distant metastasis in a series of breast tumour patients with short median follow-up (3 years).

We also identified 24 tumours with clear *maspin* overexpression, mRNA expression ranged from 3.1- to 47-fold that was found in normal breast tissue. Several authors have previously reported cases of maspin-overexpressing breast tumours (Hojo *et al*, 2001; Maass *et al*, 2001b; Umerika *et al*, 2002). By using immunohistochemical analysis, we showed that *maspin* transcripts are translated into maspin protein (Figure 1). We also showed a positive correlation between *maspin* mRNA overexpression and maspin protein abundance, and precised that alteration of *maspin* gene expression was exclusively found inside tumour epithelial cells (Figure 1). Taken together, these findings suggest that *maspin* gene expression is mainly dysregulated at the transcriptional level in breast cancer. It is also noteworthy that we found weaker maspin immunoreactivity in normal myoepithelial cells located in adjacent normal breast tissue found around all the tumour samples tested, in keeping with the literature (Sternlicht and Barsky, 1997).

In our ER*α*-positive postmenopausal breast tumour series, *maspin* mRNA overexpression was associated with poor long-term patient outcome relative to tumours with normal or low *maspin* expression (Figure 2). We also obtained evidence that maspin mRNA overexpression might be a further marker of poor outcome in patient subsets with classical markers of poor prognosis (more than three involved lymph nodes, or histopathological grade III) (Figure 3).

Primary surgery followed by adjuvant tamoxifen alone is the usual treatment for ER*α*-positive postmenopausal breast cancer. Our results, obtained in a well-defined cohort of ER*α*-positive postmenopausal breast cancer patients treated in this way, also suggest that *maspin* status, by evenly dichotomising such patients, might emerge as a useful predictor of the response to endocrine therapy. Moreover, the predictive power of *maspin* expression appears to be independent of two other genes, *ERBB2* and *ERβ*, which have been proposed to predict the response to endocrine therapy (Speirs *et al*, 1999; Stal *et al*, 2000). Indeed, we found no link between *maspin* mRNA status and the mRNA status of these two genes (Table 3). Confirmation of the predictive value of *maspin* parameter in the response to endocrine therapy in breast cancer patients needs a prospective randomised study to show that this parameter do influence the outcome only in patients who received adjuvant tamoxifen as compared with untreated patients.

This relation between *maspin* gene overexpression and poor outcome in breast cancer is in agreement with Umerika *et al* (2002), but conflicts with the reported role of *maspin* as a tumour-suppressor gene that inhibits cell migration and angiogenesis. Maass *et al* (2001c), in a series of pancreatic tumours, found that *maspin* expression was gradually upregulated with increasing malignancy, from normal pancreas tissue to precancerous lesions and invasive carcinoma. One possible explanation for these paradoxical results is that *maspin* expression is upregulated in order to neutralise for overexpression of tumour-activating gene targets. It is noteworthy in this respect that other inhibitors of tumour progression, such as PAI-1 and PAI-2 (plasminogen activator inhibitor type 1 and type 2) are overexpressed in breast cancer and associated with a poor prognosis (Stephens *et al*, 1998).

These results confirm the interest to investigate *maspin* gene expression for the detection of circulating breast tumour cells or of submicroscopic lymph-node metastases in breast cancer patients (Merrie *et al*, 1999; Sabbatini *et al*, 2000). These new informations concerning *maspin* overexpression in breast cancer associated with a poor prognosis also call into question the use of this gene or its product to inhibit angiogenesis or metastasis.

In conclusion, the results of this study point to a major role of the *maspin* gene in ER*α*-positive breast cancer. In particular, we obtained evidence that *maspin* mRNA status might serve as a new prognostic marker, and also as a possible predictor of the response to endocrine therapy in postmenopausal breast cancer patients. Finally, we describe a rapid, highly sensitive, high-throughput RT–PCR assay for determining *maspin* mRNA status.
